# A new ferroptosis-related gene model for prognostic prediction of papillary thyroid carcinoma

**DOI:** 10.1080/21655979.2021.1935400

**Published:** 2021-06-02

**Authors:** Xiaoyu Qian, Jian Tang, Lin Li, Ziqiang Chen, Liang Chen, Yongquan Chu

**Affiliations:** a Department of Head and Neck Surgery, The First Hospital of Jiaxing, the First Affiliated Hospital of Jiaxing University,Jiaxing,China; b Department of Nuclear Medicine Clinic, The First Hospital of Jiaxing, the First Affiliated Hospital of Jiaxing University, Jiaxing,China

**Keywords:** Ferroptosis, prognosis, papillary thyroid carcinoma, overall survival, gene model

## Abstract

Papillary thyroid carcinoma (PTC) is a highly heterogeneous malignancy with diverse prognoses. Ferroptosis is a new type of cell death dependent on iron. Nevertheless, the predictive ability of ferroptosis-related genes for PTC is unclear. Based on the mRNA expression information from The Cancer Genome Atlas, we compared tumor and normal tissues in terms of the gene expression, for identifying differentially expressed genes (DEGs). Then, the risk score of a 5-gene signature was calculated and a prognostic model was established to test the predictive value of this gene signature by virtue of the LASSO Cox regression. The 5 genes were validated in PTC tissues by RT-qPCR.At last, functional analysis was implemented to investigate the underlying mechanisms. We found a total of 45 ferroptosis-related genes expressed differentially between tumor and normal tissues. 6 DEGs exhibited a significant relevance to the overall survival (OS) (*P*< 0.05). We classified patients into group with high risk and group with low risk based on the median risk score of a 5-gene signature. Patients in the group with low risk presented a remarkably higher OS relative to the group with high risk (*P*< 0.01). The Cox regression analysis displayed the independent predictive ability of the risk score. The receiver operating characteristic analysis helped to validate the predictive power owned by the gene signature. After validation, the 5 genes were abnormally expressed between PTC and normal tissues. Functional analysis showed two groups had different immune status. A new ferroptosis-related gene signature can predict the outcomes of PTC patients.

## Graphical abstract

**Figure:**
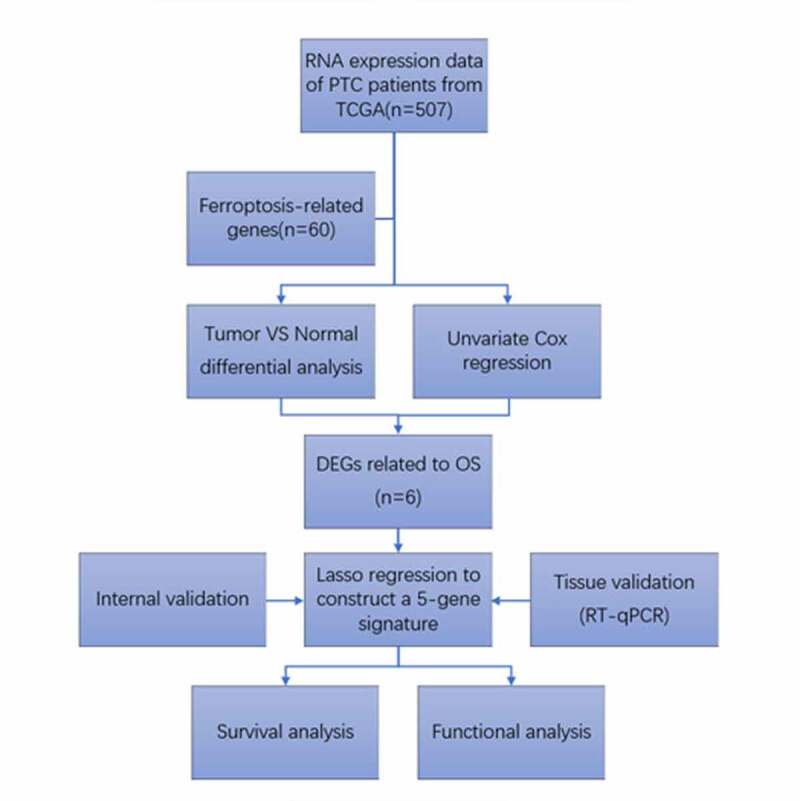

## Introduction

Papillary thyroid carcinoma (PTC) is considered the most common endocrine malignancy with multiple prognoses. The incidence of PTC has steadily increased over the last few years in many countries [1]. The survival rates of PTC patients vary greatly. While most PTC patients had an optimistic prognosis with a 10-year survival rate of 80–90%, some only had survival rates of 25% at 5-year and 10% at 10-year [2,3]. Therefore, it is necessary to develop novel predictive models for PTC.

Ferroptosis – characterized by lipid peroxidation and iron accumulation – is a form of programmed cell death [4,5]. Regulating the ferroptosis pathway has the potential to slow down cancer progression [6,7]. Studies showed that some genes, such as SLC7A11 [8], GPX4 [9] and p53 [10], regulated ferroptosis in cancer cells and affected the prognosis of hepatocellular carcinoma [11] and breast cancer [12]. However, it remains unclear whether these ferroptosis-related genes have an effect on the prognosis of PTC.

The purpose of this study was to explore the effect on the prognosis of PTC of ferroptosis-related genes and facilitate the development of prognostic models for PTC patients. We obtained PTC patients’ clinical data as well as mRNA expression information from The Cancer Genome Atlas (TCGA). The ferroptosis-related differentially expressed genes (DEGs) were used for constructing a prognostic model. Then, we validated the model. Finally, we explored the underlying mechanisms using functional enrichment analysis.

## Materials and Methods

### Data collection

The clinical data as well as the mRNA expression information regarding 507 PTC patients were obtained from the TCGA database (https://portal.gdc.cancer.gov/, up to December 2020). We used the ‘limma’ R package to normalize gene expression data. This study was exempted from ethics reviews since all data we used were publicly available, and we followed the TCGA Ethics & Policies. We selected a total of 60 ferroptosis-related genes based on previous literature [13–15].

### Construction and validation of a signature with ferroptosis-related genes

We compared gene expression levels between tumor and adjacent normal tissues to identify the DEGs using the ‘limma’ R package [16]. The criterion of DEGs was a false discovery rate (FDR) < 0.05. Cox analysis assisted in examining the ability of ferroptosis-related genes to predict overall survival (OS). Benjamini-Hochberg adjusted p-values were used to decrease FDR. The ‘glmnet’ R package served for a LASSO Cox regression – a powerful technique for variable selection and regularization – to analyze if DEGs could predict the OS and the status of the PTC patients [17]. The optimal value of the penalty parameter (λ), which corresponds to the minimum of the partial likelihood deviance, was identified by ten-fold cross-validation. We calculated the risk score using the following formula: risk score = esum (the normalized expression level regarding each gene× its regression coefficient) [11]. We then used the median risk score as the standard for classifying patients into group with high risk and group with low risk. We depicted the gene distribution in two groups by performing PCA and t-SNE using the ‘stats’ and the ‘Rtsne’ R package [18]. The ‘surv_cutpoint’ function of the ‘survminer’ R package served for the survival analysis to identify the optimal cutoff values for gene expression [19]. We then used the ‘survivalROC’ R package to perform time-dependent ROC analysis to estimate if the gene signature has predictive ability [20]. We then used the univariate and multivariate Cox regression analyses for determining if the risk score could independently predict patients’ prognosis in terms of the OS.

### Functional enrichment analysis

We conducted the Gene Ontology (GO) enrichment as well as the Kyoto Encyclopedia of Genes and Genomes (KEGG) pathway analysis of the DEGs between two groups with the ‘clusterProfiler’ R package and |log2 (fold-change)| ≥ 1 and FDR <0.05 were considered as the criteria for the DEGs [21]. The ‘gsva’ R package was applied to the single-sample gene set enrichment analysis (ssGSEA) for calculating the enrichment score regarding immune cells as well as immune-related pathways [22].

### Patients and Specimens

We selected 15 papillary thyroid carcinoma tissues and 10 normal tissues from the First Hospital of Jiaxing between 2020 and 2021. Postoperative pathological examination confirmed papillary thyroid carcinoma. This study was approved by the Ethics Committee of the First Hospital of Jiaxing. Each patient signed an informed consent. All specimens were placed in liquid nitrogen and stored at −80°C immediately.

### Quantitative Real-Time PCR

Trizol extracts total RNA from tissues and then reverse transcripts it into cDNA. PCR was performed using TB Green®Premix Ex Taq™II kit (Takara, China). The reaction process is as follows: 94°C for 30 s, 58°C for 30s, 72°C for 60 s, 40 cycles. GAPDH as internal control. The relative expression level was determined by 2− ΔΔ CT.

### Statistical analyses

Student’s t-test served for comparing the gene expressions between tumor issues and adjacent normal tissues. Chi-square test assisted in examining the differences in proportions between group with high risk and group with low risk. We performed the Mann–Whitney test for comparing the enrichment scores regarding immune cells as well as immune-related pathways between the two groups. The Kaplan–Meier method together with the log-rank test were conducted to depict the survival curves. We then conducted Cox regression analysis to figure out the factors that could predict OS. We performed all statistical analyses in R (Version 4.0.3). A two-tailed P value < 0.05 exhibited statistical significance.

## Results

In our research, we set up a prognostic model by using ferroptosis-related DEGs to investigate the effect of ferroptosis-related genes on the prognosis of PTC, and then we verified the model by internal validation and tissues validation. At last, we explored the underlying mechanisms. The results were as follows:

**Baseline data regarding** PTC **patients**

A total of 507 PTC patients were selected from the TCGA cohort whose median age was 46 (range 15–89) years. Most subjects were female and were in stage Ι. Distant metastasis occurred in a few patients. They had the median OS of 714 (range 6–5423) days. Table 1 lists the detailed characteristics of the subjects.

**Table 1.** Clinicopathologic characteristics of thyroid carcinoma patients

Characteristics	Number of case (%)
Age(years)	
<45	229 (45.2)
≥45	278 (54.8)
Median (range)	46 (15–89)
Gender	
Female	371(73.2)
Male	136(26.8)
Tumor stage	
Ι	285(56.2)
ΙΙ	52(10.3)
ΙΙΙ	113(22.3)
Ⅳ	55(10.8)
NA	2(0.4)
T stage	
T1	144(28.4)
T2	167(32.9)
T3	171(33.8)
T4	23(4.5)
Tx	2(0.4)
N stage	
N0	231(45.6)
N1	226(44.6)
Nx	50(9.8)
M stage	
M0	283(55.8)
M1	9(1.8)
Mx	214(42.2)
NA	1(0.2)
OS days (median)	714
NA- Not Available	

### Identification of ferroptosis-related DEGs with predictive value

**Figure 1.**
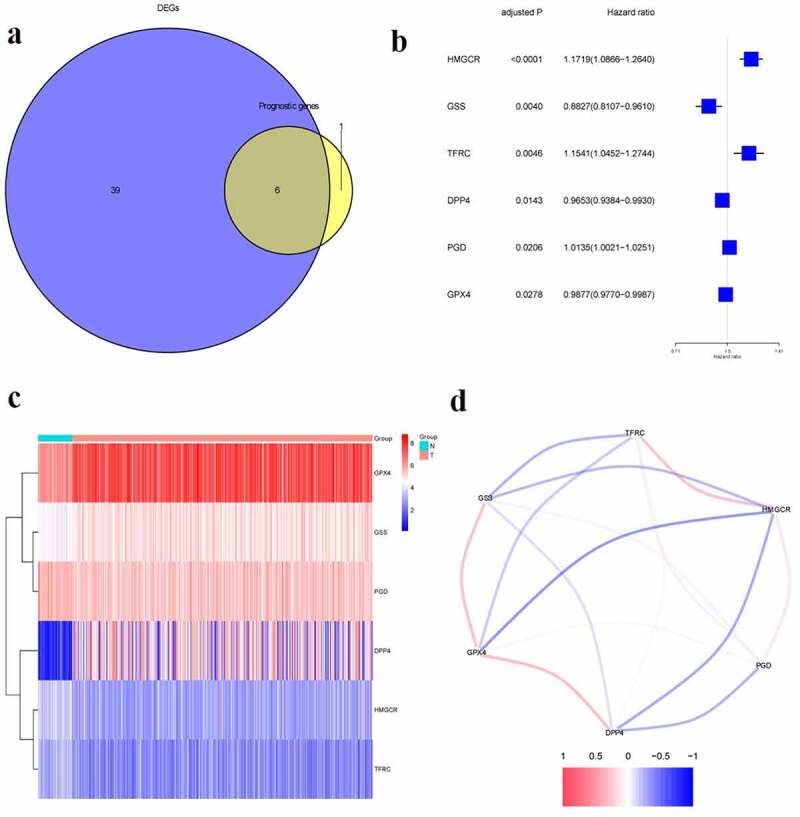
Identification of prognostic ferroptosis-related differentially expressed genes.**a**.Venn plot of the differentially expressed genes between tumor and normal tissue that were correlated with OS.**b**. Forest plot of the results of the univariate Cox regression analysis between gene expression and OS.**c**.Heatmap of the differentially expressed genes associated with OS.**d**. The correlation of the differentially expressed genes associated with OS

We found 45 ferroptosis-related genes expressed differentially between tumor and adjacent normal tissues, 6 of which presented a significant association with OS (Figure 1a, p < 0.05). The forest plots displayed the associations of gene expression with OS (Figure 1b). The heatmap indicated tumor and adjacent normal tissues could be distinguished by the DEGs (Figure 1c). Figure 1d showed the associations between these genes.

### Construction of a prognostic model

**Figure 2.**
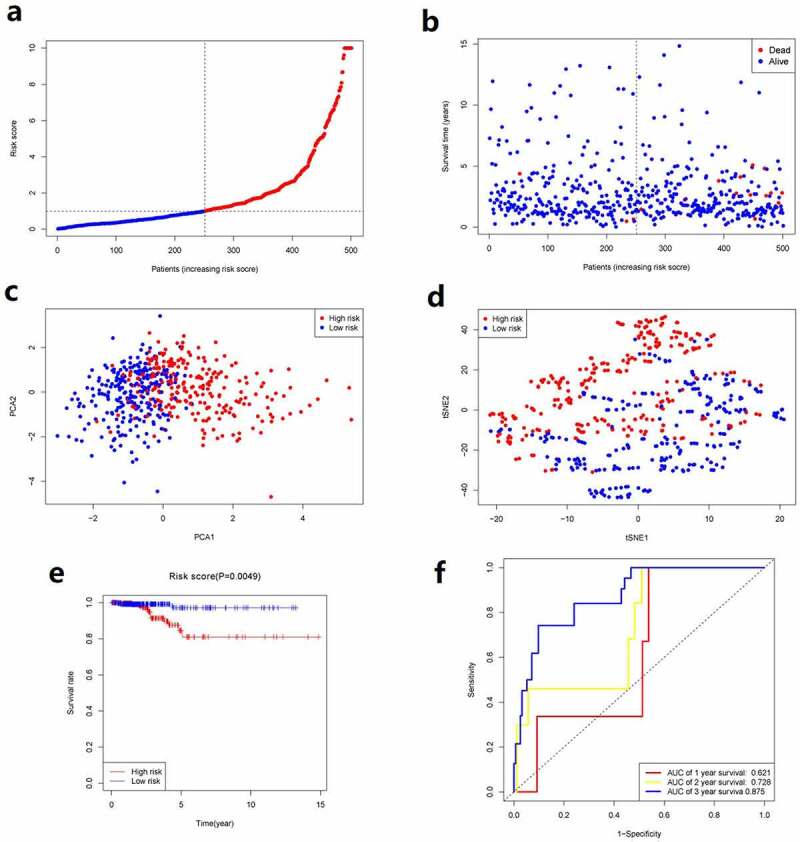
Prognostic analysis of the 5-gene signature model. **a**. The distribution and median value of the risk scores. **b**. The distributions of OS status, OS and risk score. **c**. PCA analysis of the TCGA cohort. **d**. t-SNE analysis of the TCGA cohort. **e**. Kaplan–Meier curves of the OS in the two groups. **f**.AUC of time-dependent ROC curves evaluated the prognostic capacity of the risk score

Aforementioned 6 genes were employed for establishing a prognostic model by virtue of LASSO Cox regression analysis, which were associated with OS. A 5-gene signature was then determined according to the optimal value of λ. We used the following formula to calculate the risk score: e ^(1.051 * the level of expression of HMGCR + 0.913 * the level of expression of GSS + 1.098 * the level of expression of TFRC +0.977 * the level of expression of DPP4 + 1.008 * the level of expression of PGD)^. We used the median risk score as the standard for classifying patients into group with high risk and group with low risk (as shown in Figure 2a). Between the two groups, the stages of cancer were significantly different (Table 2, *P*< 0.05). PCA and t-SNE analysis showed the patients with PTC were distributed in two directions between the two groups (Figure 2c-d). Patients in group with high risk exhibited a higher mortality rate relative to group with low risk (Figure 2b). Similarly, the Kaplan–Meier curve displayed that group with low risk had remarkably higher OS relative to group with high risk (Figure 2e, *p*< 0.05). Figure 2f showed the OS predictive power of the risk score, with the area under the curve (AUC) of 0.621, 0.728, and 0.875 at 1 year, 2 years, 3 years, respectively.

**Table 2.** Clinicopathologic characteristics of the patients in different risk groups

Characteristics	High risk	Low risk	P value
Age(%)			0.443
≥45y	142(28.3)	108(21.6)	
<45y	133(26.5)	118(23.6)	
Gender(%)			1.000
Female	183(36.5)	183(36.5)	
Male	67(13.4)	68(13.6)	
Tumor stage(%)			0.002
I+ II	185(36.9)	148(29.5)	
III+IV	64(12.8)	102(20.4)	
unknown	1(0.2)	1(0.2)	
T stage			<0.001
T1+ T2	175(34.9)	131(26.1)	
T3+ T4	74(14.8)	119(23.8)	
unknown	1(0.2)	1(0.2)	
N stage			<0.001
N0	140(27.9)	89(17.8)	
N1	74(14.8)	148(29.5)	
Unknown	36(7.2)	14(2.8)	
M stage			0.475
M0	134(26.7)	148(29.5)	
M1	5(1.0)	4(0.8)	
unknown	111(22.2)	99(19.8)

### Independent predictive ability of the risk score

**Figure 3.**
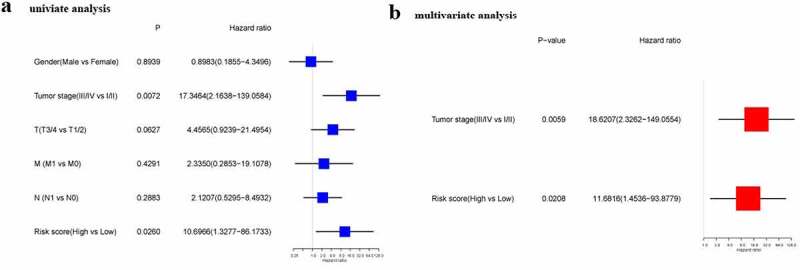
Results of univariate and multivariate Cox regression analysis on OS

Cox regression analysis served for confirming if the risk score could predict OS independently. As found by the univariate Cox regression analysis, the risk score exhibited a significant association with OS (HR = 10.697, 95% CI = 1.328–86.173, P = 0.026) (Figure 3a). Consistently, the multivariate Cox regression analysis discovered a significant association between the risk score and OS (HR = 11.682, 95% CI = 1.454–93.878, P = 0.021) (Figure 3b).

### Functional analysis in the TCGA

**Figure 4.**
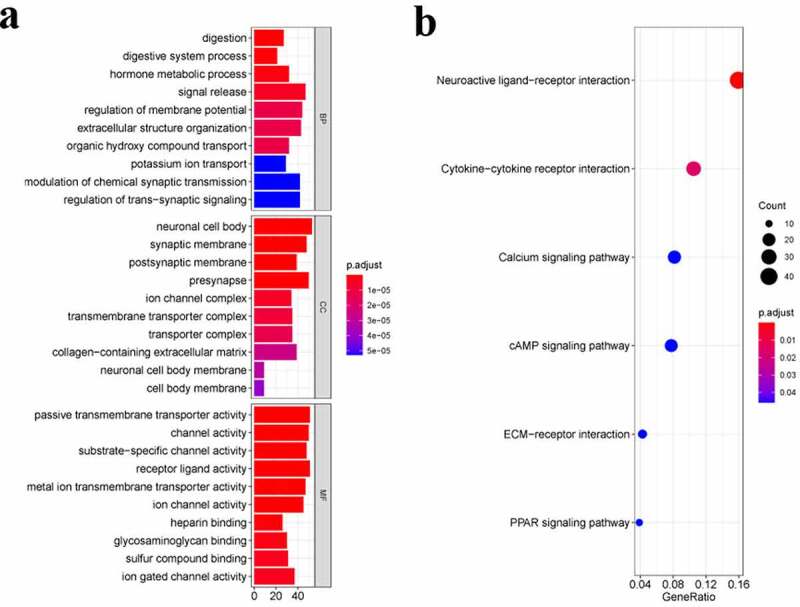
Functional enrichment analysis of DEGs. **a**. Top 10 biological process (BP) terms, cellular components (CC) terms, molecular functions (MF) terms. **b**. Top 6 Kyoto Encyclopedia of Genes and Genomes (KEGG) pathways

We conducted the GO enrichment as well as KEGG pathway analysis of the DEGs for identifying the biological functions and pathways related to the risk score. Figure 4a showed the top 10 BP terms, CC terms, and MF terms. The main enriched Go terms were digestion, neuronal cell body, synaptic membrane, passive transmembrane transporter activity, and channel activity. Figure 4b showed the top 6 KEGG pathways, namely the neuroactive ligand–receptor interaction, the ytokine-cytokine receptor interaction, the cAMP signaling pathway, the ECM–receptor interaction, and the PPAR signaling pathway.

**Figure 5.**
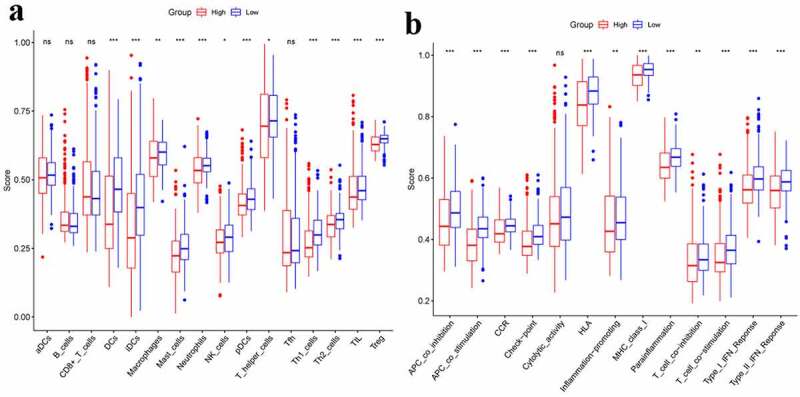
Comparison of the ssGSEA scores between different risk groups.**a**. The scores of 16 immune cells.**b**.The scores of 13 immune-related functions. Adjusted P values were showed as: ns, not significant; *, *P*< 0.05; **,*P*< 0.01; ***, *P*< 0.001

The immune status was quantified by ssGSEA using the enrichment score and its association with the risk score was analyzed. Between two groups, the enrichment scores of Treg, TIL, Th1-cells, T-helper-cells, pDCs, NK-cells, Neutrophils, Mast-cells, Macrophages, IDCs, and DCs were significantly different (adjusted *P*< 0.05, Figure 5a). The two groups presented an obvious difference in terms of scores of MHC-class-I, Parainflammation, HLA, CCR, T-cell-co-stimulation, T-cell-co-inhibition, Inflammation-promoting, APC-co-stimulation, APC-co-inhibition, Check-point, Type-I-IFN-Response, as well as Type-II–IFN-Response (adjusted *P*< 0.05, Figure 5b).

### Internal validation of the 5-gene signature

**Figure 6.**
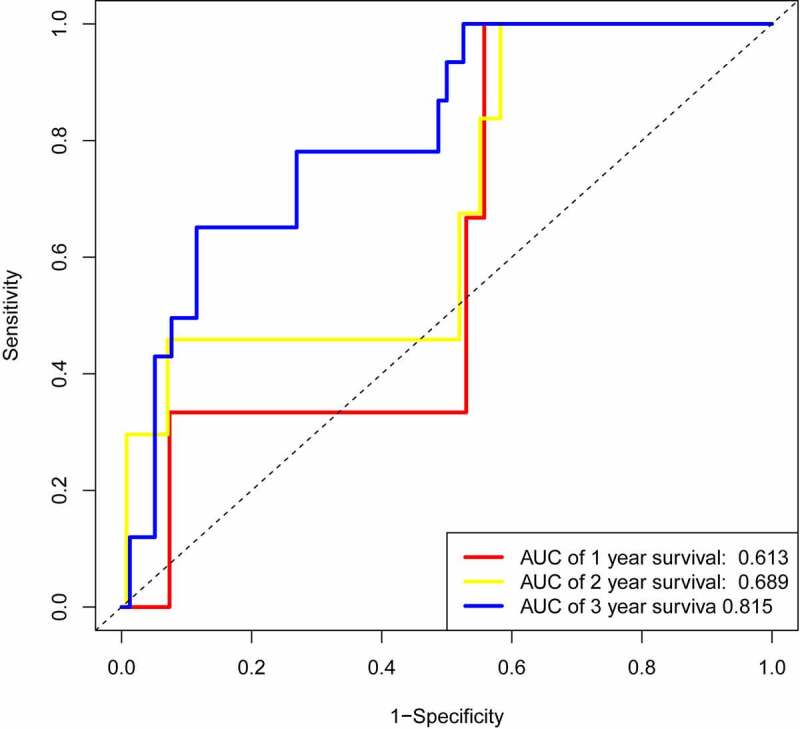
AUC of time-dependent ROC curves of the internal validation

We randomly selected half of the data to perform time-dependent ROC analysis for internal validation, the results showed that the area under the curve (AUC) of 0.613, 0.689, and 0.815 at 1 year, 2 years, 3 years, respectively (Figure 6), which were close to our results (Figure 2f) .

### Validation of the 5 ferroptosis-related genes in PTC tissues

**Figure 7.**
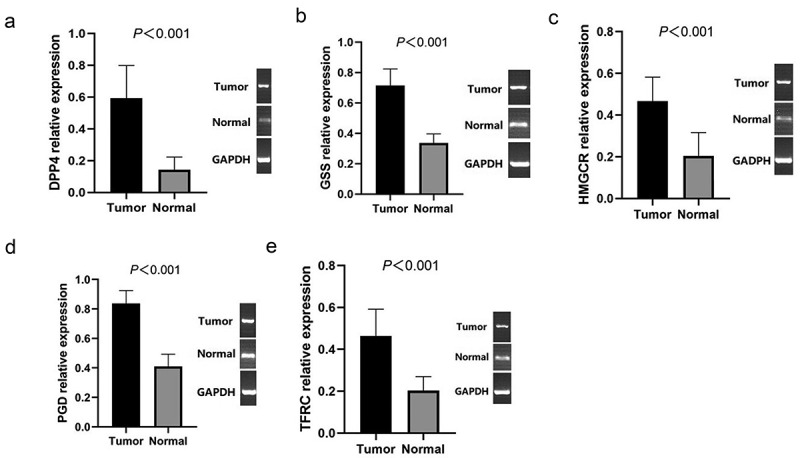
RT-qPCR detecting the mRNA expression levels of DPP4 (a), GSS (b), HMGCR (c), PGD (d), TFRC (e) in PTC and normal tissues

We further validated the mRNA expression levels of the 5 ferroptosis-related genes in PTC tissues and normal tissues by RT-qPCR, the results showed that DPP4 (Figure 7a), GSS (Figure 7b), HMGCR (Figure 7c), PGD (Figure 7d), TFRC (Figure 7e) were all significantly higher in PTC tissues than in normal tissues (*P*< 0.001).

## Discussion

Ferroptosis was first proposed by Dixon [4] in 2012 and was crucial in the occurrence and development of many tumors. Previous studies [23,24] found several ferroptosis-related genes may be involved in PTC. However, their associations with the OS of PTC patients remained unknown. In this research, we explored the correlation between 60 ferroptosis-related genes and the prognosis of PTC. We further constructed a new predictive model with 5 genes related to ferroptosis.

In our study, most ferroptosis-related genes (75%, 45/60) expressed differentially between tumor and adjacent normal tissues and six genes were related to the OS, which implied ferroptosis was involved in PTC and showed the possible predictive value of these genes.

We used 5 ferroptosis-related genes (DPP4, GSS, HMGCR, PGD, TFRC) to construct a predictive model. These genes act through different mechanisms [7,13]. DPP4, which is involved in glutathione and amino acid metabolism, promotes lipid peroxidation through NOXs. Ferroptosis could be limited by the p53 by blocking DPP4 activity in colorectal cancer cells [25]. GSS is the target gene of NRF2, which promotes resistance to ferroptosis [26]. The inhibition of HMGCR results in enhancing FIN-56-induced ferroptosis and blocking mevalonic acid synthesis [27]. PGD acts through pentose phosphate pathway and is an important regulator in tumor cells. In non-small-cell lung cancer (NSCLC) cells Calu-1, knockdown of PGD inhibits erastin-triggered ferroptosis [27]. For iron metabolism, silencing TFRC can inhibit erastin-triggered ferroptosis [28]. Similarly, knockdown of TFRC inhibits ferroptosis caused by the deprivation of amino acid/cystine [29,30].To sum up, three of the genes (DPP4, PGD, TFRC) in the prognostic model can accelerate ferroptosis, while the other two genes (GSS, HMGCR) can protect cells from ferroptosis. Some studies [12,31] reported that DPP4 was upregulated in PTC patients and TFRC was associated with unfavorable prognoses. In our study, all the 5 ferroptosis-related genes were upregulated among PTC patients and were related to unfavorable clinical outcomes. However, whether these genes affecting the prognosis of PTC through regulating ferroptosis deserves further research.

As the mechanisms of ferroptosis in tumors was still elusive, we conducted GO enrichment and KEGG pathway analysis. We found digestion, neuronal cell body, synaptic membrane, passive transmembrane transporter activity, and channel activity were enriched, which provide a new direction for future research. Interestingly, the higher-risk group had remarkably higher contents of macrophages and Treg cells than group with low risk in this study. Considering tumor-associated macrophages [32,33] and Treg cells [34] are significantly related to cancer cell migration and poor prognoses in PTC patients, the unfavorable clinical outcomes in group with high risk might be caused by the impaired antitumor immunity.

Admittedly, our prognostic model was limited by using public databases. It is necessary to conduct more in vitro and in vivo studies for verifying the clinical application value owned by this model.

## Conclusion

Our research identified a new predictive model using 5 ferroptosis-related genes. The risk score could predict the OS of PTC independently. The underlying mechanism of the association of the ferroptosis-related genes and PTC is unclear and deserves further study.

## Data Availability

The data analyzed in the study can be found on the TCGA database (https://portal.gdc.cancer.gov/).
